# Successful resection of a huge metastatic liposarcoma in the pericardium resulting in improvement of diastolic heart failure: a case report

**DOI:** 10.1186/s40792-015-0079-4

**Published:** 2015-09-02

**Authors:** Yoshiyuki Yamashita, Kazuhiro Kurisu, Satoshi Kimura, Yasutaka Ueno

**Affiliations:** Department of Cardiovascular Surgery, Shimonoseki City Hospital, 1-13-1 Koyo-cho, Shimonoseki, Yamaguchi 750-8520 Japan

**Keywords:** Liposarcoma, Pericardial metastasis, Diastolic heart failure

## Abstract

Although liposarcoma often metastasizes to various organs, cardiac metastasis, including to the pericardium, is rare. We present a case of a third recurrence of pericardial metastasis from the thigh, which required surgical resection because of cardiac failure. Surgery was effective for improving cardiac function and reintroducing chemotherapy. This is the first reported case of metastatic pericardial liposarcoma, which was successfully resected three times. We believe that aggressive surgical treatment, when it can resolve cardiac impairment, potentially leads to a more favorable prognosis.

## Background

Liposarcoma is one of the most common soft tissue sarcomas in adults and often develops in the retroperitoneum and lower limbs. This tumor usually metastasizes to different organs, but metastasis to the heart, including to the pericardium, is rare. We report a patient with repeated cardiac metastases that required surgical resection.

## Case presentation

A 66-year-old woman was referred to our hospital because of recurrence of pericardial metastatic liposarcoma with progressive dyspnea. The patient had undergone surgical resection of a primary liposarcoma in the left thigh 19 years previously and resection of a locally recurrent tumor 13 years previously. She developed cardiac and pericardial metastasis 10 years later. Resection of a cardiac metastasis originating from the right ventricle was performed via median sternotomy 3 years previously, and a second resection followed by radiotherapy for recurrence of the pericardium metastasis adjacent to the diaphragm 2 years previously. Each of these metastatic tumors was large enough to result in cardiac failure. The pathological diagnosis of the resected primary and metastatic tumors was myxoid liposarcoma. The third recurrence of metastatic pericardial liposarcoma was detected 1 year previously and was followed by metastasis to the posterior mediastinum, liver, retroperitoneum, and peritoneum. Chemotherapy including gemcitabine, docetaxel, pazopanib, and epirubicin was administered for 1 year. Chemotherapy was effective for all of the metastases except those in the pericardium and posterior mediastinum. As the pericardial metastasis enlarged, the patient developed dyspnea over a period of several months. Her symptoms were speculated to be due to diastolic impairment by the large tumor. Reintroduction of chemotherapy was difficult because of cardiac failure. Therefore, she was referred to our hospital for surgical treatment of the pericardial metastasis.

**Fig. 1:**
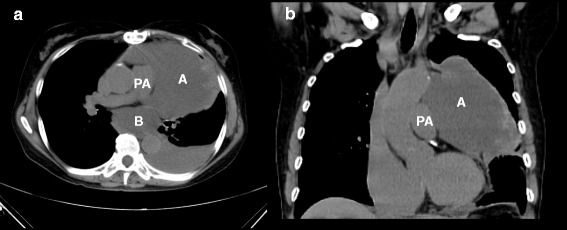
Preoperative computed-tomography images. Computed tomography of **a** horizontal dislocation and **b** coronal dislocation showing a large mass in the pericardium anterolateral to the main pulmonary artery (*A*). Horizontal dislocation also showed a mass in the posterior mediastinum posterior to the bronchi (*B*). *PA* pulmonary artery

**Fig. 2:**
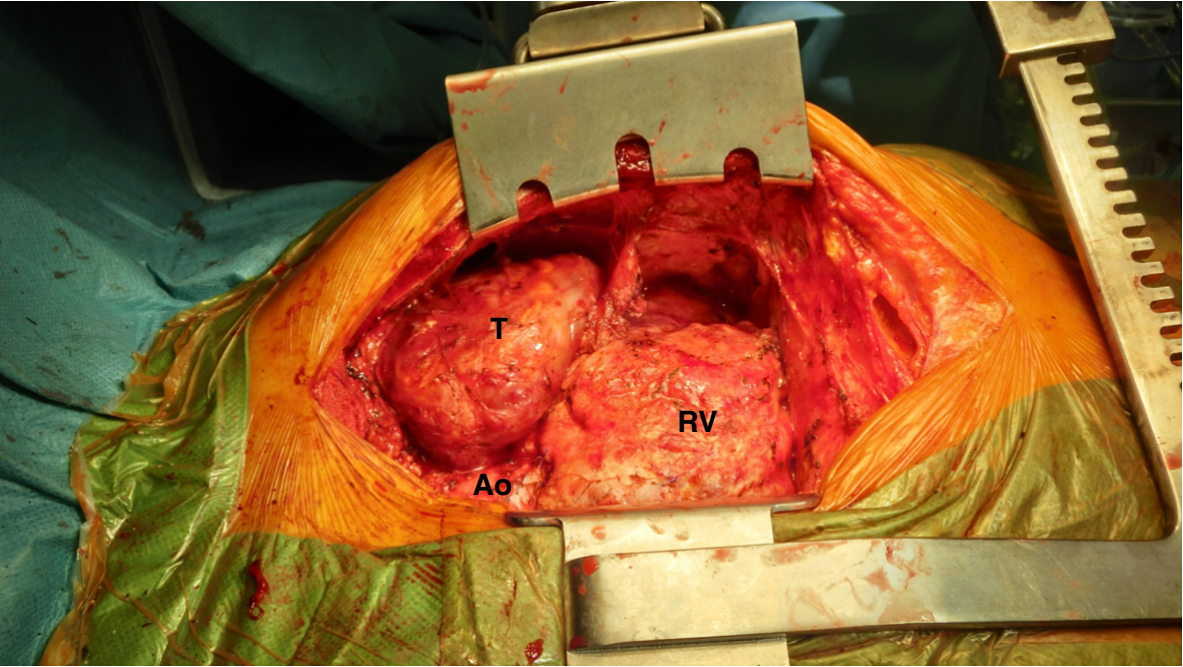
Intraoperative picture. A large tumor can be seen anterolateral to the main pulmonary artery (*T*). *Ao* ascending aorta, *RV* right ventricle

**Fig. 3:**
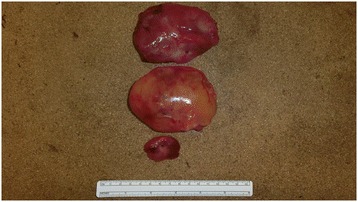
Picture of the en bloc resected tumors. Two large masses were anterolateral to the main pulmonary artery, and one small mass was adjacent to the right ventricle. All of them were soft and mucinous

**Fig. 4:**
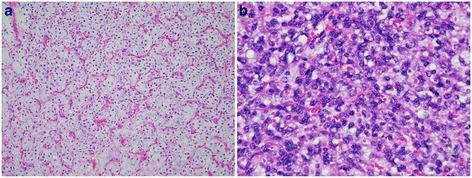
Histological findings of the resected tumors. Histological findings showing that almost all of the tumors were myxoid liposarcomas (**a**, hematoxylin-eosin, ×100) and a part of the tumor adjacent to the right ventricle was round-cell liposarcoma (**b**, hematoxylin-eosin, ×200)

**Fig. 5:**
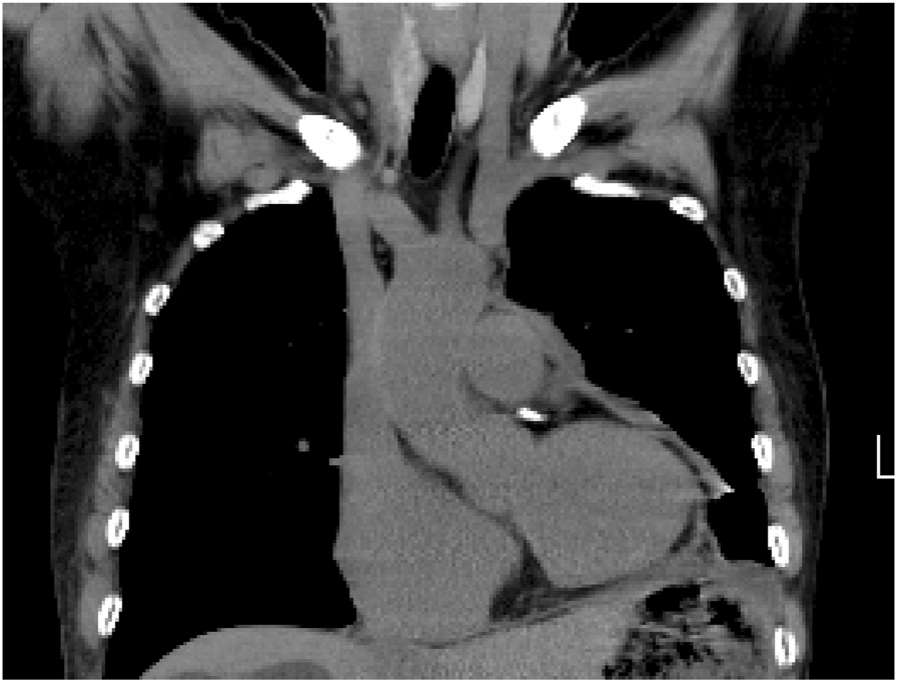
Postoperative computed-tomography images. Computed tomography showed that the pericardial tumor was almost totally resected and there was no evidence of recurrence

On admission, the patient had moderate dyspnea. Signs of cardiac failure, including engorgement of the jugular vein and edema of the lower extremities, were observed. Laboratory data showed an elevated brain natriuretic peptide level of 524 pg/ml. Chest X-ray showed a large mass lateral to the left ventricle extending to the left chest wall. Echocardiography showed a mass lateral to the main pulmonary artery in the pericardium. The left ventricular ejection fraction was preserved (60.9 %), but the inferior vena cava was dilated. Computed tomography showed a large, mostly low-density mass in the pericardium. This mass extended laterally to the ascending aorta and aortic arch, anterolaterally to the main pulmonary artery, and anteriorly to the left pulmonary artery (Fig. 1). The mass was speculated to be partially involved in the pulmonary artery. Masses were also identified in the posterior mediastinum, liver, retroperitoneum, and peritoneum. At the time of cardiac catheterization, the right atrial pressure and pulmonary capillary wedge pressure were elevated (11 and 17 mmHg, respectively), and simultaneous measurement of the right and left ventricular pressure demonstrated elevated and nearly equal end-diastolic pressure (16 and 18 mmHg, respectively). The patient’s hemodynamic data corresponded to diastolic impairment. Accordingly, resection of the pericardial tumor was recommended. We performed a median re-sternotomy along the previous skin incision. Moderate adhesion was present between the heart and chest wall, and we safely exposed the right ventricle, right atrium, and ascending aorta (Fig. 2). A mucinous mass measuring approximately 30 × 20 × 10 mm, which was not recognized before the operation, was observed between the diaphragm and right ventricle and was resected (Fig. 3). The mass and involved pericardium were easily detached from the main and left pulmonary arteries and ascending aorta, and we did not need to use cardiopulmonary bypass. The mass comprised three parts, all of which were mucinous and measured approximately 100 × 50 × 20 mm each. We performed en bloc resection of two of the three masses (Fig. 3), but the other was vulnerable and disintegrated. Therefore, we removed them as much as possible. The total weight of the masses was 610 g. The pathological diagnosis of the large masses was myxoid liposarcoma, and that of the smaller mass was myxoid and round-cell liposarcoma (Fig. 4). The patient had an uneventful postoperative course, and her symptoms markedly improved. Cardiac catheterization showed a decreased pulmonary capillary wedge pressure of 10 mmHg. Two months later, she underwent resection of the posterior mediastinal metastasis, which had become larger in spite of the chemotherapy, to reduce the tumor volume. This was performed via video-assisted left posterolateral thoracotomy. Chemotherapy was then reintroduced, and the patient was in good condition 3 months after the cardiac surgery. Computed tomography 3 months after the cardiac surgery showed no evidence of recurrence in the pericardium (Fig. 5).

### Discussion

Liposarcoma is the second most common malignancy of soft tissues. The most common primary sites of liposarcoma are the retroperitoneum and lower limbs. Although liposarcoma often metastasizes to different organs, cardiac metastasis, including to the pericardium, is rare. Thirty-five cases of metastatic cardiac liposarcoma have been reported in the literature [1–6], with only nine cases of pericardial metastases [2–4]. According to the WHO classification, liposarcoma is divided into the following categories: well-differentiated, differentiated, myxoid, round-cell, pleomorphic, mixed-type liposarcoma, and liposarcoma, not otherwise specified. Myxoid and well-differentiated liposarcomas have more favorable 5-year survival rates than round-cell and pleomorphic types. The first choice of therapy for primary liposarcoma is surgical resection. However, local recurrence or distant metastasis often occurs even many years after surgery. Radiotherapy combined with surgery may be associated with less recurrence [7]. Chemotherapy is an option in the case of metastatic or unresectable disease [8], but it is still an empirical decision owing to the lack of evidence. Pericardial metastasis of liposarcoma often manifests as diastolic cardiac impairment. Surgical resection of cardiac or pericardial metastasis is usually recommended as a potentially radical treatment for solitary lesions without any other metastasis and is occasionally adopted as a palliative procedure in patients with other metastases. In our case, the patient had symptoms of cardiac failure because of impaired diastolic filling caused by the pericardial metastasis. She could not undergo chemotherapy because of cardiac failure. Resection of the tumor was expected to relieve the diastolic impairment and provide a chance of reintroduction of chemotherapy, which could have potentially prolonged her prognosis. Therefore, we decided to perform a third intervention, and the strategy was effective. This is the first reported case of metastatic pericardial liposarcoma, which was successfully resected three times. The prognosis of patients with cardiac metastatic liposarcoma is usually poor. Aoyama et al. [9] reported 15 cases of metastatic cardiac liposarcoma; six patients died within 6 months, and the longest follow-up was 15 months. However, the follow-up period of our patient from the first resection of the pericardial metastasis was 51 months. To the best of our knowledge, this is the longest reported follow-up of pericardial metastasis of liposarcoma. Aggressive surgical treatment with the intention of resolving the cardiac impairment may provide a symptom-free interval, and even a prolonged prognosis.

## Conclusions

We successfully resected a pericardial metastatic liposarcoma, resulting in symptom improvement and reintroduction of chemotherapy. Aggressive surgical therapy may provide a symptom-free interval and prolonged prognosis.

## Consent

Written informed consent was obtained from the patient for publication of this case report and any accompanying images. A copy of the written consent is available for review by the editor in chief of this journal.
